# Pregnant mothers have limited knowledge and poor dietary diversity practices, but favorable attitude towards nutritional recommendations in rural Ethiopia: evidence from community-based study

**DOI:** 10.1186/s40795-018-0251-x

**Published:** 2018-12-20

**Authors:** Taddese Alemu Zerfu, Sibhatu Biadgilign

**Affiliations:** 1 Maternal and Child Well Being Unit, African Population and Health Research Center, Nairobi, Kenya; 2 Dilla University, College of Medicine and Health Sciences and Referral Hospital, Addis Ababa, Ethiopia; 3 Center for Food Science and Nutrition, College of Natural Sciences, Addis Ababa University, Addis Ababa, Ethiopia; 4 Public Health and Nutrition Research Consultant, P.O. Box 24414, Addis Ababa, Ethiopia

**Keywords:** Attitude, Ethiopia, Dietary diversity, Knowledge, Nutrition, Pregnant mother, Pregnancy

## Abstract

**Background:**

Mothers’ nutrition is crucial for good pregnancy outcomes and in improving children’s nutritional status. The present study aimed to examine the level of knowledge and attitude towards maternal nutrition and dietary diversity practices among pregnant mothers in rural central Ethiopia.

**Methods:**

In-depth analysis of data from a prospective study involving a total of 389 eligible pregnant women, enrolled during their second antenatal care (ANC) visit was conducted between August 2014 and March 2015. Study participants were selected by employing systematic sampling techniques. Dietary diversity practices were assessed by asking each individual pregnant woman to provide a single 24-h dietary recall. Simple frequencies and graphs were used to present the analyzed data and interpretations.

**Results:**

Vegetables were listed top as major sources of vitamin A (45.5%) and iron (23.8%). Nearly half (47%) of the mothers lacked awareness on balanced and diversified diets. Conversely, nearly three fourths (73.8%) and two thirds (66.8%) of them had favorable attitudes towards dietary diversity and early initiation of antenatal care follow up. With a median dietary diversity score of four, starchy staples (100%), legumes and nuts (89.2%) were major food groups consumed by almost all of the mothers included in the study.

**Conclusion:**

Though pregnant mothers had limited knowledge and poor dietary diversity practices, they exhibited a relatively favorable attitude towards major nutritional recommendations. Use of antenatal care and its follow up as a point of entry for educating pregnant women and increasing nutrition knowledge and attitude is recommended.

## Background

Adequate and quality maternal nutrition is important for the health and reproductive performance of women as well as the health, survival, and development of children [1, 2]. Particularly, the first 1000 days of life, from conception to age two, is often considered as “the window of opportunity to prevent chronic malnutrition, childhood obesity and medical complications arising later in life” [3, 4]. Improvements in maternal and child nutrition can reduce multiple risks of Adverse Pregnancy Outcomes (APO) such as: fetal growth restriction, low birth weight babies and small-for-gestational age births. It also helps to prevent micronutrient deficiencies [5, 6]. As the high nutritional cost of pregnancy and lactation contributes significantly to their poor nutritional status, healthy nutrition intake during pregnancy is critical for pregnant women [7].

In Ethiopia, even if the right to adequate nutrition is enshrined and tremendous nutrition interventions have been undertaken, nutritional problems and infectious diseases are still amongst the major health problems of the country [8, 9]. Cognizant to this, evidences from the country and other similar settings shows that high under nutrition rates prevail, even among regions and households where food is plenty [9]. Nutritional knowledge and attitude are important factors of dietary practices and are, thus, potential targets for appropriate planning of nutrition interventions for vulnerable population groups like those lactating and women that are pregnant [10, 11]. Nutrition education enhances nutritional knowledge, thereby influencing attitude and practices towards good nutrition [12, 13]. Mother’s knowledge of nutrition is a very important nexus in good pregnancy outcomes and a crucial skill in improving children’s nutritional status [11].

Evidence on maternal knowledge and attitudes towards nutrition during pregnancy, and its association with their dietary diversity practices is hardly available. Existing studies [11, 14] reported maternal awareness levels or association with some pregnancy outcomes but lacked explanations of how awareness levels relate to attitudes and dietary diversity practices during pregnancy. Therefore, in this study, we aimed to assess the level of maternal knowledge and attitude towards nutrition during pregnancy and their relations with dietary diversity and nutritional practices of pregnant mothers in rural central Ethiopia.

## Methods

### Study design and setting

A cross-sectional, multi-center survey, with relevant nutritional and health data was collected from pregnant mothers attending antenatal care (ANC) in selected health centers, between August and December 2014 in Arsi Zone, Oromia region, of Ethiopia. The Arsi Zone is located in the central part of Ethiopia.

### Sample size determination and sampling procedure

Sample size for the study was calculated using Epi-info 7 statistical software considering the following parameters: a power of 80%, a confidence interval of 95%, and a prevalence of 64.4% of pregnant mothers with adequate knowledge of nutrition during pregnancy [14]. In addition, a 10% non-response rate was considered. he minimum sample size required for the study was set to be 389 pregnant mothers.

Initially, we estimated the total number of pregnant women in a health center per annum (half a year in this case) and an average of this was considered for taking the sample. Accordingly, local annual reports showed that about 23 new pregnant mothers were enrolled to antenatal care services per month per health center. This has yielded a total of 184 pregnant mothers per month, which in turn resulted in a total of 1104 mothers in a six-month period in the study health facilities. Systematic sampling techniques were employed to identify the 389 mothers. A sample interval of three was calculated by dividing total study mothers throughout the entire period by the samples required. Therefore, data collecting nurses enrolled one in every three mothers attending antenatal care until an adequate number of samples was reached.

### Data collection methods and measurement procedures

Data was collected between August 2014 and March 2015 in rural health facilities (health posts and health centers) and homes of mothers coming from nearby villages, depending on the preferences of mothers and conduciveness. Data was collected using a pre-tested and standardized interviewer administered questionnaire. The data collection tool consisted of items on the respondents’ socio demographic characteristics, knowledge on components of nutrition with direct and significant effect on pregnancy, attitude towards key nutritional recommendations, dietary intake or dietary diversity practices and other nutritional variables. Nutritional components with direct effects on pregnancy include vitamin A, iron, and iodine. Accordingly, mothers were asked for knowledge on food items which are sources of these nutrients without probing (just by recall). Mothers were declared not to know about the nutrient if they reported “Don’t know”. This was of course evaluated thoroughly by explaining and re-explaining the questions to the mother and final decisions were made if the mother failed to know either the nutrients at all or the food sources.

Attitude was assessed by asking scenarios that reflect the item of interest. In this regard, five items were considered as key themes of assessment including, dietary diversity, nutritional values of foods, weight gain during pregnancy, decreased workload, and timing for antennal care. After presenting the scenarios designed on each item, mothers were asked to agree or disagree with the ideas of those presented in the scenario.

Dietary diversity and nutritional practices during pregnancy were assessed by asking each individual pregnant woman using single 24-h dietary recall, which were used to calculate their dietary diversity scores (DDS). In order to include a community’s dietary characteristics and seasonality of food consumption, all days of the week, except holidays as well as three (out of the four) major seasons were covered in the data collection. Food items reported by each pregnant woman were categorized into nine groups according to the latest Food and Agriculture Organization of the United Nations (FAO) guidelines [15].

### Anthropometric measurement

The anthropometric measurements for the study participants were performed using the standardized approach recommended by The World Health Organization (WHO) [16]. The participant’s weight was measured to the nearest 100 g on electronic scales. Similarly, height was measured to the nearest mm (SECA 206 Body Meter). The mid-upper arm circumference (MUAC) of the pregnant women was taken in the left arm and measured to the nearest mm. No women with unreliable measurements due to a physical handicap were found and anthropometric measures were taken for all mothers.

### Field workers, training, and supervision

The data collectors were midwives working in the maternal and child health (MCH) units of each health center after receiving intensive training on how to enroll and collect relevant data. All of the midwives were fluent speakers of the local language, Afan Oromo as well as Amharic. Trained research assistants and principal investigators (PI) supervised the data collection process, provided onsite technical support for the data collectors, and checked all the completed questionnaires daily. Training, field-testing, and standardization of measurements were carried out before the actual fieldwork. Morning meetings, led by the principal investigator, were held with all field workers throughout the data collection period and continuous concrete feedback was provided on the spot.

### Statistical analysis

Double data entry was performed with Epi-data statistical software. Data quality was maintained by quality checks during both data collection and data entry. Further cleaning was also considered during data management. All statistical analyses were carried out using Statistical Package for the Social Sciences (SPSS) Statistics (version 20.0). Simple frequencies were run, and tables and graphs were created to present descriptive data. Chi-square test was used to test for independence distribution of categorical variables (socio-demographic characteristics, categorized nutritional indices, and dietary intake) between health centers and districts.

## Results

### Socio-demographic characteristics of the mothers

A total of 374 pregnant mothers gave full and complete responses yielding a response rate of 96.1%. The mean (+/− SD) age of respondents was 29.98 (+/− 4.46) years, ranging between 19 and 37 years. Only 16.8% of the mothers included in the study were aged above or equal to 30 years. Educationally, over half (57.5%) of the students were either illiterate or were able to read or write only and lacked formal education. Religious affiliation of respondents were as follows: majority (52.9%) of were followers of the Orthodox Tewahido Church followed by Muslims (38.8%). In the same way, a large majority (80.5%) were Oromo by ethnic background followed by Amhara’s (13.9%). Almost all (94.7%) were married with few (3.7%) divorced by martial status (Table 1).

**Table 1:** Socio-demographic characteristics of pregnant mothers (*n*=314) in central Arsi, rural Ethiopia

Socio-demographic variable	Number	Percent
Age groups		
• 15 - 19	34	9.1
• 20 - 24	147	39.3
• 25 - 29	130	34.8
• 30 +	63	16.8
Maternal educational status		
• No formal Education	120	32.1
• Primary Education	95	25.4
• Primary and above	92	24.6
Maternal religion		
• Orthodox	198	52.9
• Muslim	145	38.8
• Protestant	31	8.3
Maternal marital status		
• Married	354	94.7
• Divorced	14	3.7
• Widowed	6	1.6
Family size		
• 1 - 3persons	224	59.9
• 4 -6 persons	132	35.3
• ≥ 7 persons	18	4.8
• Mean +/- SD	3.56	2.4
Land Size		
• <1 Hectares	136	36.4
• 1 - 2 Hectares	70	18.7
• 2+ Hectares	168	44.9

### Anthropometric measurements of the study participants

Anthropometric assessment results showed that the mean (+/− SD) height, weight and MUAC of pregnant women participating in the study was 156.7 (+/− 5.32) cm, 52.3 (+/− 6.74) Kg and 22.7 (+/− 1.61) cm, respectively. Less than one in twenty (3.2%) and one in ten (11.4%) had a height of 150 cm and a weight of 45 k or less respectively. Contrarily, nearly half (46.3%) had a MUAC of < 23 cm (Table 2).

**Table 2:** Anthropometric measurements of the of pregnant mothers (*n*=314) in central Arsi, rural Ethiopia

Anthropometric Characteristic	Number	Percent
Maternal height		
< 150 cm	12	3.2
> = 145 cm	362	96.8
Mean +/-SD	158.68	5.32
Maternal weight		
< 45 kg	42	11.4
> =45 kg	326	88.6
Mean +/-SD	52.29	6.74
Maternal MUAC		
< 23 cm	173	46.3
≥ 23 cm	201	53.7
Mean +/-SD	22.72	1.61

### Awareness of mothers about food sources of key nutrients (vitamin a and iron)

**Fig. 1:**
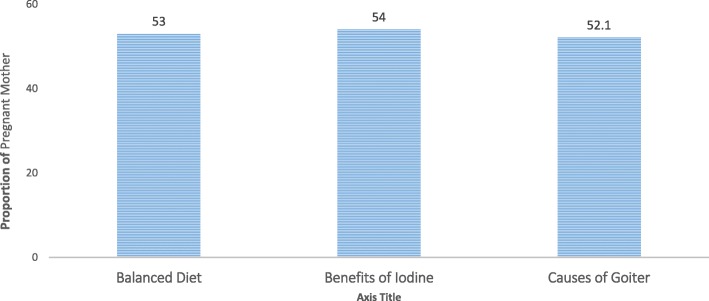
Knowledge of pregnant mothers about balanced diet and iodine in rural Arsi, central Ethiopia

Among mothers who reported to know food sources of vitamin A and iron, in both cases, a majority (45.5% and 23.8%) mentioned vegetables followed by wheat (16%), barley (15.2%) and fruits (13%) as sources of for vitamin A. Similarly, teff (16.8%), egg (13.9%) and milk (12.3%) were among the most frequently mentioned sources of iron. Conversely, the latter was mentioned least (5.4% and 1.3%) by either, and close to half (46.8%) and one third (33.4%) mentioned that they have no idea about any of the food sources for iron and vitamin A. In the same way, nearly half (47%) reported lack of awareness about the meanings of balanced diets and dietary diversity among food groups (Table 3). Figure 1 shows the knowledge of pregnant mothers about balanced diet and the benefit or the effects of iodine on human health.

**Table 3:** Knowledge of pregnant mothers about food sources of vitamin A and iron; in central Arsi, rural Ethiopia

Food items	Vitamin A Sources	Iron Sources
	Number (%)	Number (%)
Teff	39 (10.4)	63 (16.8)
Wheat	60 (16)	20 (5.3)
Barely	57 (15.2)	37 (9.9)
Vegetables	170 (45.5)	89 (23.8)
Fish	57 (15.2)	28 (7.5)
Meat	48 (13)	37 (9.9)
Egg	65 (17.7)	52 (13.9)
Milk	47 (12.8)	46 (12.3)
Fruit	51 (13)	29 (7.8)
Oil/butter	31 (8.4)	10 (2.7)
Salt	20 (5.4)	5 (1.3)
Don’t know	123 (33.4)	175 (46.8)

**Fig. 2:**
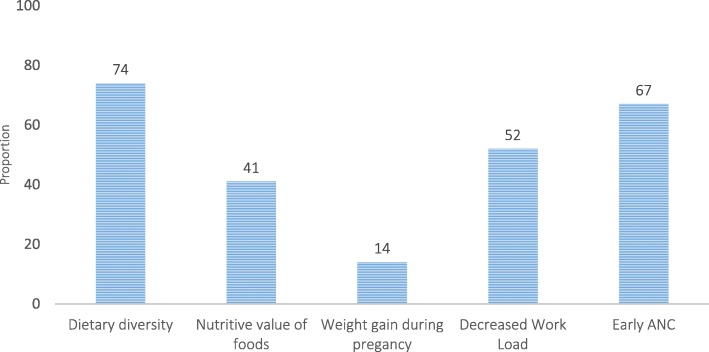
Proportion of pregnant mothers with favorable attitude towards key nutrition and health care recommendations during pregnancy, Arsi zone, central Ethiopia

Close to three in four (73.8%) mothers had favorable attitudes towards dietary diversity and early initiation of antenatal care (66.8%) and follow up for better pregnancy and pregnancy outcomes. Greater than one in ten (13.7%) disfavored weight gain during pregnancy. Other nutritional and health care recommendations during pregnancy, including consumption of nutrient rich foods and decreasing workload during pregnancy were favored modestly (Fig. 2).

### Dietary diversity practices

**Fig. 3:**
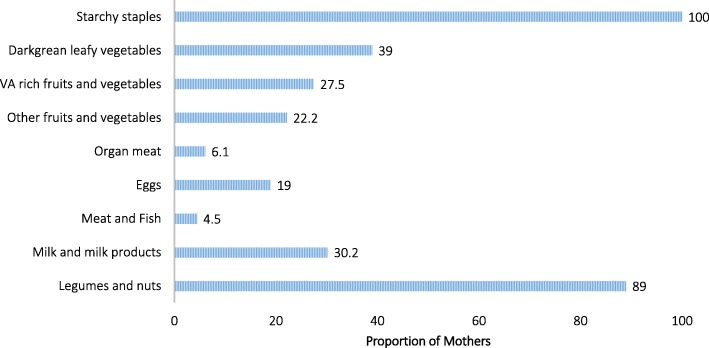
Dietary diversity patterns and food groups consumed by pregnant women in rural Arsi, central Ethiopia

The mean (+/− SD) and median dietary diversity score of pregnant mothers was 3.57 (+/− 1.67) and 4 respectively. Starchy staples, legumes and nuts were major and dominant food groups as consumed by all (100%) and (89%) of respondents. Nonetheless, animal source foods were least consumed. Only (7%) and (5%) percent of the study participants reported consumption of organ meat and fish. Green leafy vegetables, milk & milk products, and fruits and vegetables were consumed by a small proportion of mothers in descending order (Fig. 3).

### Association of maternal nutrient food sources with dietary diversity practices

Compared to those who had non-diversified diet, pregnant mothers who had diversified diet, frequently mentioned vegetables (53% Vs 34.4%) and fish (20.4% Vs 9%) as major food sources of vitamin A (*P* < 0.05). Conversely, mothers who had less diversified diet more frequently mentioned wheat (20.3% Vs 11%) or declared to know none (48.5% Vs 28.1%) of the food source of vitamin A compared to the diversifying mothers (*P <* 0.05), Table 4. Similarly, wheat (8.3% Vs 1.5%) and egg (19.3% Vs 9.8%) were more frequently mentioned food sources of iron by pregnant mothers who had diversified diet compared to non-diversified (*P* < 0.05), Table 5. Otherwise, no statistically significant difference was observed in all other food sources mentioned as sources of either vitamin A or iron.

**Table 4:** Association between knowledge of vitamin A food sources with maternal dietary diversity during pregnancy in central Arsi, rural Ethiopia

Vitamin A Food Sources mentioned	Dietary Diversity		*P*-value
	Non-diversified Number (%)	Diversified Number (%)	
Teff			
Yes	10 (7.8)	19 (10.5)	0.24
No	123 (92.5)	162 (89.5)	
Wheat			
Yes	27 (20.3)	20 (11)	0.01^*^
No	106 (79.7)	161 (89)	
Barely			
Yes	20 (15)	25 (13.8)	0.44
No	113 (85)	156 (86.2)	
Vegetables			
Yes	46 (34.6)	96 (53)	0.001^*^
No	87 (65.4)	85 (47)	
Fish			
Yes	12 (9)	37 (20.4)	0.04^*^
No	121 (91)	144 (79.6)	
Meat			
Yes	15 (11.5)	22 (12.4)	0.48
No	115 (88.5)	156 (87.6)	
Egg			
Yes	27 (20.8)	25 (14)	0.08
No	103 (79.2)	153 (86)	
Milk			
Yes	13 (10)	27 (15.2)	0.12
No	117 (90)	151 (84.8)	
Fruits			
Yes	15 (11.5)	31 (17.4)	0.10
No	115 (88.5)	147 (82.6)	
Salt			
Yes	10 (7.7)	7 (3.9)	0.12
No	120 (82.3)	171 (96.1)	
Don't know any VA food source			
Yes	63 (48.5)	50 (28.1)	0.00^*^
No	67 (51.5)	128 (71.9)	

*p < 0.05

**Table 5:** Association between knowledge of iron food sources with maternal dietary diversity practices during pregnancy in central Arsi, rural Ethiopia

Iron food sources mentioned	Dietary Diversity		*P*-value
	Non-diversified Number (%)	Diversified Number (%)	
Teff			
Yes	12 (9)	36 (19.9)	0.086
No	121 (91)	145 (80.1)	
Wheat			
Yes	2 (1.5)	15 (8.3)	0.006^*^
No	131 (98.5)	166 (93.7)	
Barely			
Yes	12 (9)	20 (11)	0.35
No	121 (91)	161 (89)	
Vegetables			
Yes	27 (20.3)	40 (22.1)	0.405
No	106 (79.7)	141 (77.9)	
Fish			
Yes	8 (6)	15 (8.3)	0.29
No	125 (94)	166 (91.7)	
Meat			
Yes	12 (9)	21 (11.6)	0.29
No	121 (91)	160 (88.4)	
Egg			
Yes	13 (9.8)	35 (19.3)	0.014^*^
No	120 (90.2)	146 (80.7)	
Milk			
Yes	17 (12.8)	21 (11.6)	0.44
No	116 (87.2)	160 (88.4)	
Fruits			
Yes	6 (4.5)	17 (9.4)	0.075
No	127 (95.5)	164 (90.6)	
Salt			
Yes	2 (1.5)	2 (1.1)	0.565
No	131 (85.5)	179 (89.9)	
Don't know any VA food source			
Yes	63 (48.5)	50 (28.1)	0.12
No	67 (51.5)	128 (71.9)	

*p < 0.05

### Association of maternal attitude towards maternal nutrition and dietary diversity practices

Cognizant to awareness levels, mothers who reported to have diversified diet had better favorable attitude (45.9% Vs 33.8%) towards nutritive value of food compared to those who had non-diversified diet (*P <* 0.05). However; attitude towards dietary diversity and better and adequate nutrition during pregnancy didn’t showed statistically significant difference between these groups (*P* > 0.05), Table 6.

**Table 6:** Association between attitude towards maternal nutrition and dietary diversity practices of pregnant mothers in central Arsi, rural Ethiopia

Attitude component	Dietary Diversity		*P*-value
	Non-diversified number (%)	Diversified number (%)	
Dietary diversity			
• Favorable	33 (25.6)	45 (25)	0.36
• Unfavorable	93 (72.1)	135 (75)	
Nutritive value of foods			
• Favorable	44 (33.8)	83 (45.9)	0.02^*^
• Unfavorable	86 (66.2)	98 (54.1)	
Nutrition during pregnancy			
• Favorable	57 (43.8)	93 (51.4)	0.11
• Unfavorable	73 (56.2)	88 (48.6)	

*p < 0.05

## Discussion

Using data from an existing cohort study, the present study aimed to assess the level of awareness and maternal attitudes towards nutritional recommendations and dietary diversity practices during pregnancy. Pregnant mothers from rural villages were interviewed in private and separate settings according to preference, either at a nearby health facility or home, giving responses of their experience and knowledge about nutrition during pregnancy. Findings of the analysis revealed that the overall awareness of mothers about food sources and benefits of key micronutrients during pregnancy (iron and vitamin A) and dietary diversity or a balanced diet was limited. Mothers had poor dietary diversity and nutritional care practices, however attitudes were favorable towards both.

Assessing their knowledge about the food sources of vitamin A and iron, a sizable proportion of the mothers reported to know some food items that are available to their vicinity in both cases. Vegetables being mentioned frequently as major sources of both iron and vitamin A.

Scientifically; even if vegetables are rich in vitamin A, they contain the inactive carotenoids (Provitamin), which the body must convert to the active retinoid which are obtained solely from animal source foods like meat and milk. Unless people consume the active retinol forms of vitamin A, mere consumption of provitamins can’t guarantee availability of the nutrient [17]. This could be the main reason for high levels of vitamin A deficiency rates in the country, putting it among serious public health problems [18]. Despite in the study area where plant-based food sources are staple diets, the prevalence of vitamin A deficiency is very high, as people mainly rely on cereal grains, and other plant-based food sources which contain non-active forms of some of the micronutrients including vitamin A rich foods [19]. In related context, vegetables are also not as such iron sources. This rather could indicate a widespread misconception about the sources of iron in the community. On the other hand, about 13–16% of respondents mentioned that fruits; grains like wheat, barley, teff; and some animal source foods like egg and milk are the main sources of iron. This continue to show lack of clear and proper understanding or confusions about the food sources of iron in the community. Evidence suggests consuming red meat, such as beef, is particularly recommended as an important source of iron.

In both cases, it seems there is a widespread misconception and poor knowledge about the food sources of major nutrients during pregnancy. This low level of awareness and misperception about the food sources of iron is consistent with findings of previous and similar studies in Ethiopia [14, 20–22] and elsewhere [23, 24]. One of the studies in Ethiopia indicated that close to three-fourths (74.0%) of respondents were not aware of the main food groups or having a balanced diet. The same study indicated that an increasing awareness and knowledge provides information which may stimulate changing of attitude and subsequently result in the enhancement of healthy dietary practices [14]. Studies from other high-income countries also indicate that nutritional knowledge of mothers during pregnancy is sub-optimal. According to a Malayan study, only (65.7%) of the participants were able to give correct responses to questions related to nutritional knowledge [25].

Misconceptions and lack of awareness about nutrition in general and maternal nutrition in particular could be a main reason for low dietary diversity and proper nutritional care during pregnancy leading to high nutritional problems and their adverse consequences. Studies from other similar low-income settings also showed that pregnant women have inadequate intake of iron, folate, fruits, and vegetables, related to poor knowledge [26].

Promisingly and unconditional to existing facts that knowledge is a structural property of attitudes or a function of the number of beliefs and experiences linked to the attitude in memory and the strength of the associative links, we found a much higher favorable attitude towards the practice of balanced diets and dietary diversity food groups. In the current analysis, half (47%) of the mothers were unaware of what to eat during pregnancy and the meaning of a balanced diet and dietary diversity, however, attitudes and opinions of a balanced diet were promising.

Within high income settings, nutritional knowledge was found to be positively associated with education, household income, vitamin/mineral supplementation and regular physical activity [27]. Other studies have also indicated that, above-average nutritional knowledge was independently associated with the use of iron-folic acid and multivitamins [11]. In a Kenyan study, (46%) of the women in the study had a moderate level of nutrition knowledge, (44.6%) had a moderate health knowledge level, and (80.7%) had a moderate DDS level [28]. Similarly, a study from South Western Ethiopia, indicated that between half to three-fourths of mothers were aware of foods and food groups during pregnancy [14].

In our present analyses, unlike the low level of awareness about nutrition and dietary sources of iron and vitamin A, a considerable proportion of mothers had favorable attitudes towards dietary diversity (73.8%) and early initiation of antenatal care (66.8%) for better pregnancy and outcomes. This is contrary to theoretical behavioral models [29] and several study findings [30, 31]. Likewise, studies demonstrated that women with a higher level of education and had more than 10 prenatal visits were more likely to use iron supplements during pregnancy.

Analysis of association between maternal knowledge of food sources of selected micronutrients (vitamin A and iron) with dietary diversity practices of mothers showed that mothers who diversified their diets mentioned vegetables and fish as key food sources. On the other hand, wheat was more frequently mentioned as key food source of vitamin A. This shows that the former group is not only diversifying, but also more knowledge about the correct food sources of vitamin A. Mothers with less diversified group; on the other hand, were mentioning non-correct food items as well as unknowledgeable about food sources. This was also reflected in their attitude towards nutritive value of foods.

The present study had some limitations that need to be taken into consideration when interpreting the findings. Although it is recognized that both pre- and early-pregnancy nutrition are associated with pregnancy outcomes, we were only able to enroll women in their second trimester, mainly because of the late start of ANC visits. Furthermore, in spite of our efforts to collect data from all women during pregnancy, we didn’t collect data from those who were not visiting health facilities for antenatal care, which could potentially mask the views and awareness levels of those not attending. Our study was a health-facility based study and hence might have favored those with better access to health facilities.

## Conclusion

Notwithstanding the above limitations, our study highlights the fact that the overall awareness level of pregnant mothers on nutrition during pregnancy, particularly their knowledge about sources of key micronutrients (iron and vitamin A) and a balanced or diversified diet was limited. Cognizant to this, the practices related to proper and diversified nutrition during pregnancy were also found to be poor. Nonetheless, a promising and favorable attitude towards dietary diversity and nutritional care practices were observed. This indicates that there is a conducive and great potential to ensure maternal dietary diversity and proper nutritional practices during pregnancy, even with modest, but targeted awareness raising activities along with economic empowerment of women to ensure access to necessary food items.

## Abbreviations

ANC: Antenatal Care
APO: Adverse Pregnancy Outcomes
DDS: Dietary Diversity Scores
FAO: Food and Agriculture Organization of the United Nations
MCH: Maternal and Child Health
MUAC: Mid-Upper Arm Circumference
PI: Principal Investigator
SD: Standard deviation
SPSS: Statistical Package for the Social Sciences
WHO: World Health Organization

## References

[1] Bloomfield F.H., Spiroski Ana-Mishel, Harding J.E. (2013). Fetal growth factors and fetal nutrition. Seminars in Fetal and Neonatal Medicine.

[2] Nabarro David (2013). Global child and maternal nutrition—the SUN rises. The Lancet.

[3] Pem D. Factors Affecting Early Childhood Growth and Development: Golden 1000 Days. Adv Practice Nurs. 2015;1:101. 10.4172/2573-0347.1000101.

[4] Mameli Chiara, Mazzantini Sara, Zuccotti Gian (2016). Nutrition in the First 1000 Days: The Origin of Childhood Obesity. International Journal of Environmental Research and Public Health.

[5] Khan Ashraful Islam (2013). Effects of pre- and postnatal nutrition interventions on child growth and body composition: the MINIMat trial in rural Bangladesh. Global Health Action.

[6] Imdad A, ZA B (2011). Effect of balanced protein energy supplementation during pregnancy on birth outcomes. BMC Public Health.

[7] McGowan C a, McAuliffe FM (2012). Maternal nutrient intakes and levels of energy underreporting during early pregnancy. Eur J Clin Nutr.

[8] Central Statistical Agency (CSA) [Ethiopia] and ICF. Ethiopia Demographic and Health Survey 2016. Addis Ababa: CSA and ICF.

[9] Ferede A, Lemessa F, Tafa M, Sisay S (2017). The prevalence of malnutrition and its associated risk factors among women of reproductive age in Ziway Dugda district, Arsi zone, Oromia regional state, Ethiopia. Public Health.

[10] Perumal N, Cole DC, Ouédraogo HZ, Sindi K, Loechl C, Low J, et al. Health and nutrition knowledge , attitudes and practices of pregnant women attending and not-attending ANC clinics in Western Kenya: a cross-sectional analysis. BMC Pregnancy Childbirth. 2013:1–12. 10.1186/1471-2393-13-146 PMC3716969 23845074

[11] Popa AD, Niţă O, Graur Arhire LI, Popescu RM, Botnariu GE, Mihalache L (2013). Nutritional knowledge as a determinant of vitamin and mineral supplementation during pregnancy. BMC Public Health.

[12] Shariff ZM, Bukhari SS, Othman N, Hashim N, Ismail M, Kasim SM (2008). Nutrition education intervention improves nutrition knowledge , attitude and practices of primary school children : a pilot study. Health Educ.

[13] Girard Amy Webb, Olude Oluwafunke (2012). Nutrition Education and Counselling Provided during Pregnancy: Effects on Maternal, Neonatal and Child Health Outcomes. Paediatric and Perinatal Epidemiology.

[14] Daba G, Beyene F, Fekadu H, Garoma W. Assessment of Knowledge of Pregnant Mothers on Maternal Nutrition and Associated Factors in Guto Gida Woreda, East Wollega Zone, Ethiopia. J Nutr Disorders Ther. 2013;4:130. 10.4172/2161-0509.1000130.

[15] Kennedy G, Ballard T, Dop MC. Guidelines for Measuring Household and Individual Dietary Diversity. Food and Agriculture Organization of the United Nations (FAO) 2013. ISBN 978-92-5-106749-9.

[16] WHO (1995). Physical Status : The use and interpretation of anthropometry.

[17] Haskell MJ (2012). The challenge to reach nutritional adequacy for vitamin a: β-carotene bioavailability and conversion - evidence in humans. Am J Clin Nutr.

[18] Demissie T, Ali A, Mekonen Y, Haider J, Umeta M (2010). Magnitude and distribution of vitamin a deficiency in Ethiopia. Food Nutr Bull.

[19] Demissie T, Ali A, Mekonnen Y, Haider J, Umeta M (2009). Demographic and health-related risk factors of subclinical vitamin a deficiency in Ethiopia. J Health Popul Nutr.

[20] Gebremedhin S, Samuel A, Mamo G, Moges T, Assefa T (2014). Coverage, compliance and factors associated with utilization of iron supplementation during pregnancy in eight rural districts of Ethiopia: a cross-sectional study. BMC Public Health.

[21] Alderman H. Child Malnutrition in Ethiopia: Can Maternal Knowledge Augment The Role of Income? Child Malnutrition in Ethiopia : Can Maternal Knowledge Augment The Role of Income? 2001;(22).

[22] Zerfu TA, Umeta M, Baye K (2016). Dietary habits, food taboos, and perceptions towards weight gain during pregnancy in Arsi, rural Central Ethiopia: a qualitative cross-sectional study. J Health Popul Nutr.

[23] Ajantha SAK, Malhotra B, Mohan SK, Joshi A (2015). Evaluation of dietary choices, preferences, knowledge and related practices among pregnant women living in an Indian setting. J Clin Diagnostic Res.

[24] Bwibo NO, Neumann CG (2003). Animal source foods to improve micronutrient nutrition and human function in developing countries: Th need for animal source foods by Kenyan children. J Nutr.

[25] Pon LW, Noor-Aini MY, Ong FB, Adeeb N, Seri SS, Shamsuddin K (2006). Diet, nutritional knowledge and health status of urban middle-aged Malaysian women. Asia Pac J Clin Nutr.

[26] Nowicki E, Siega-Riz A-M, Herring A, He K, Stuebe A, Olshan A (2011). Predictors of measurement error in energy intake during pregnancy. Am J Epidemiol.

[27] Worsley A (2002). Nutrition knowledge and food consumption-can nutrition knowledge change behaviour?. Asia Pac J Clin Nutr.

[28] Ekesa BN, Walingo MK, Abukutsa-Onyango M (2009). Dietary diversity, nutrition status and morbidity of pre-school children in Matungu division, Western Kenya. Int J Food Safety Nutr Public Heal.

[29] Bendoly E, Croson R, Goncalves P, Schultz K (2010). Bodies of knowledge for research in behavioral operations. Prod Oper Manag.

[30] Elhassan MR, Gamal HE, Mohammed GSS (2013). Nutrition knowledge attitude and practices among students of Ahfad University for women. Indian J Sci Res.

[31] Sun P-C, Huang H-L, Chu F-Y (2015). Factors instead of demographic characteristics related to nutrition label use. Br Food J.

